# Evaluation of *CYP2C9*- and *VKORC1*-based pharmacogenetic algorithm for warfarin dose in Gaza-Palestine

**DOI:** 10.4155/fsoa-2017-0112

**Published:** 2018-01-10

**Authors:** Basim Mohammad Ayesh, Ahmed Shaker Abu Shaaban, Abdalla Asaf Abed

**Affiliations:** 1 Department of Laboratory Medical Sciences, Faculty of Applied Sciences, Alaqsa University, Gaza, Palestine; 2 Abdul Aziz al Rantisi Paediatric Hospital Laboratory, Ministry of Health, Gaza, Palestine; 3 Biology Department, Faculty of Science, Islamic University of Gaza, Gaza, Palestine

**Keywords:** allelic frequency, *CYP2C9*, Gaza strip, IWPC algorithm, pharmacogenetics, vitamin K antagonist, *VKORC1*, warfarin

## Abstract

**Aim:**

To evaluate applicability of CYP2C9*2, *3 and *VKORC1–1639G > A* based algorithm to predict warfarin stable dose (WSD) in a group of Palestinian patients.

**Patients & methods:**

Warfarin doses were retrospectively calculated for 101 Palestinian patients under warfarin therapy using three models. Performance of the three models was assessed in 47 patients found to take WSD.

**Results:**

Frequency of CYP2C9*2, *3 and *VKORC1–1639G > A* alleles is 13.6, 0.0 and 46.5% respectively. The international warfarin pharmacogenetics consortium algorithm was more reliable (MAE = 8.9 ± 1.4; R^2^ = 0.350) than both the clinical algorithm (MAE = 10.4 ± 1.4; R^2^ = 0.128;) and the fixed-dose algorithm (MAE = 11.1 ± 1.7).

**Conclusion:**

The international warfarin pharmacogenetics consortium algorithm can be reliably applied for predicting the WSD in Palestinian population.

## Lay abstract

The aim of this study was to evaluate applicability of an algorithm that is based on the patient genotype to predict the warfarin stable dose in a group of Palestinian patients. The international warfarin pharmacogenetics consortium algorithm was more reliable than both a clinical algorithm and a fixed-dose algorithm. The international warfarin pharmacogenetics consortium algorithm can be reliably applied for predicting the warfarin stable dose in Palestinian population.

Warfarin is a vitamin K antagonist used as the standard oral anticoagulant for venous and arterial thromboembolic disorders. A patient on warfarin therapy is at continuous risk for thrombosis or bleeding because of multiple environmental and genetic factors [1–6]. Among the key genetic causes of variability in response to warfarin are polymorphisms in the cytochrome P450, family 2, subfamily C, polypeptide 9 (*CYP2C9*) and the vitamin K epoxide reductase complex 1 (*VKORC1*) genes [7–13]. The CYP2C9*2 allele (c.430 C > T; p.Arg144Cys; rs1799853) and *3 allele (c.1075A > C; p.Ile359Leu; rs1057910) are believed to dominantly impair the metabolism of S-warfarin by 30–40% and 80–90%, respectively [10]. Furthermore, lower requirement of warfarin maintenance dose is associated with impaired *VKORC1* expression because of a prevalent promoter single nucleotide polymorphism (c.-1639 G > A) [12,14]. Therefore, the average daily warfarin doses differ by race/ethnicity [15,16]. The international warfarin pharmacogenetics consortium (IWPC) has recommended an algorithm for estimating the initial warfarin weekly dose based on *CYP2C9* and *VKORC1* genotypes and some patient's clinical and anthropometric measures [17]. The algorithm has been recommended by the latest guidelines for warfarin treatment of a different range of conditions [17,18].

In Gaza strip, warfarin is empirically administered to a wide range of patients with repeated dose adjustment until the target INR is reached. A 5 mg/day warfarin dose is initially administered followed with a number of INR tests and dose adjustments. Attempts to reach a WSD are accompanied with a high risk of bleeding adverse effects. To the best of our knowledge, warfarin therapy has never been assessed in Gaza strip, neither has the prevalence of *CYP2C9* and *VKORC1* allelic variants been determined. Therefore, in this study we aimed at determining the applicability of the IWPC pharmacogenetic based algorithm for prediction of warfarin dose, depending on *CYP2C9* and *VKORC1* alleles among Palestinian population of Gaza strip.

## Materials & methods

### Patients & sample collection

The study population was recruited from the anticoagulation clinics of the European Gaza Hospital and Al-Shifa Hospital during a 3-month period. The recruited patients were under warfarin therapy.

Approximately, 2.5 ml of venous blood were collected in EDTA tube from each participant and processed for genomic DNA extraction. Another 1.8 ml peripheral blood were collected in tubes containing 3.2% sodium citrate for prothrombin time (PT) and INR testing. Patients were assured for vitamin K free diet at least three days before blood sample collection.

### PT & INR measurement

Sodium citrate blood specimens were centrifuged at 1600–2000 × *g* for 10 min to obtain platelet poor plasma. Prewormed 100 μl of the plasma or controls were incubated with prewormed 200 μl thromboplastin reagent (Dutch Diagnostics, Netherlands) at 37°C. The time required for clot formation was recorded in seconds and the INR was calculated using the formula (INR = (PT_patient_/PT_normal_)^ISI^), where PT_normal_ is the mean value for normal patients in seconds (15.0 s as calculated in the study) and ISI is the International Sensitivity Index for the thromboplastin reagent (1.03 as set by the manufacturer). The targeted therapeutic range of INR was set at (2.0–3.0) for every patient except for three patients who have had mechanical heart valve replacement and needed their INR to fall between 2.5 and 3.5, as clinically recommended [19].

### DNA extraction & PCR/RFLP detection of CYP2C9*2, CYP2C9 *3 & *VKORC1* (1639G > A) allelic variants

Genomic DNA was extracted from 300 μl peripheral blood leukocytes using the Wizard Genomic DNA Extraction Kit (Promega, USA) according to the manufacturer recommendations.

The deficiency allele CYP2C9*2 was analyzed by amplification of a 454 bp fragment in exon 3 flanking the nucleotide substitution site, and *Ava*II restriction digestion [20]. The PCR reactions were carried out in 25 μl volumes containing 2 μl of extracted DNA, 10 pmol of each primer (5′-GTATTTTGGCCTGAAACCCATA-3′ in forward direction and 5′-GGCCTTGGTTTTTCTCAACTC-3′ in reverse direction) and 1× GoTaq Green master mix (Promega, USA). The cycling profile consisted of an initial denaturation step at 94°C for 5 min; followed by 35 cycles of 94°C for 60 s, 52°C for 45 s and 72°C for 60 s and a 10-min final extension at 72°C. The resulting 454 bp product was digested for 1 h with 10 U/μl *Ava*II at 37°C in the provided buffer and analyzed by electrophoresis in a 2.5% agarose gel. *Ava*II cuts the PCR products of the wild-type allele into 397 and 57 bp fragments.

The null allele CYP2C9*3 was analyzed, similar to CYP2C9*2, by amplifying a 105 bp fragment from exon 7 flanking the nucleotide substitution site and *Kpn*I restriction digestion [21]. The primer sequences were (5′-TGCACGAGGTCCAGAGGTAC-3′ in forward direction and 5′-ACAAACTTACCTTGGGAATGAGA-3′ in reverse direction). The resulting 105 bp product was digested for 1 h with 10 U/μl *Kpn*I in the provided buffer at 37°C and analyzed by electrophoresis on a 3.5% agarose gel. *Kpn*I cuts PCR products containing the mutant allele into 85 and 20 bp fragments.

For detection of the *VKORC1–*1639G > A deficiency allele, the PCR reactions were carried out in 20 μl volumes containing 2 μl of extracted DNA, five pmol of each primer [22] (5′-GCCAGCAGGAGAGGGAAATA-3′ in forward direction and 5′-AGTTTGGACTACAGGTGCCT-3′ in reverse direction) and 1× GoTaq Green master mix. The cycling profile consisted of an initial denaturation step at 94°C for 5 min; followed with 30 cycles of 94°C for 60 s, 52°C for 60 s, and 72°C for 60 s; and a 5-min final extension at 72°C. The resulting 290 bp product was digested for 16 h with 20 U/μl *Msp*I in the provided buffer at 37°C and analyzed by electrophoresis in a 3.5% agarose gel. *Msp*I cuts the PCR products of the wild-type allele into 167 and 123 bp fragments.

### Data analysis

Personal and clinical data were available for 99 participants and included the weekly dose of warfarin, duration under warfarin treatment, change in warfarin dose, warfarin-associated hemorrhage. Bleeding events were classified as minor (mild nosebleeds, bruise, microscopic hematuria, and mild gingival, conjunctival or anal bleeding) and major (gross hematuria, gastrointestinal bleeding requiring medical evaluation or blood transfusion) if they occurred at least once. The data were obtained from the medical record or from the patient himself.

The INR data were used to determine if the patient has imperially reached a WSD, in other words the patient remained on the same dose for at least three consecutive visits and had a target INR within the target range. Three warfarin dose prediction models were examined in terms of their ability to predict the WSD for each patient. The IWPC pharmacogenetic algorithm (PGx) depends on *CYP2C9* and *VKORC1* genotypes and other patients’ anthropometric measures (age, height, weight, race, taking enzyme inducer and amiodarone) [17]. The clinical algorithm utilizes the same variables as the PGx model, but without the incorporation of genetic variables. The third model is based on a fixed-dose of 35 mg/week.

To compare model performance, first, the mean absolute error (MAE) was determined by calculating the average of the absolute values of the differences between the WSD and the doses predicted by each model. Second, the coefficient of determination (R2) was calculated for each model using linear regression. Finally, the percentage of patients whose predicted warfarin dose was within 20% of the empirically reached stable therapeutic dose (Ideal) was determined. Similarly, the percentage of patients with predicted dose of at least 20% higher than the actual dose (overestimation) or at least 20% lower than the actual dose (underestimation) was also calculated. These values represent a difference of 1 mg per day relative to the traditional starting dose of 5 mg per day, a difference clinicians would be likely to define as clinically relevant [23]. The benefit of using a PGx algorithm over other algorithms was further verified with the number needed to genotype (NNG) analysis. The NNG is the average number of patients who need to be genotyped for one of them to benefit compared with a control. It is defined as the reciprocal of the absolute risk reduction (ARR), which is calculated as the absolute difference between the event rate (ER) for the PGx algorithm and the ER for the compared algorithm (clinical or fixed-dose). ER the probability of a bad event in other words (underestimation + overestimation)/total number of patients. The ideal NNG is one, and the higher the NNG, the less effective is the treatment.

Data were tabulated and analyzed using the Statistical Package for Social Science software version 17. The accuracy and completeness of all questions was assured by frequency distribution and cross tabulation for the entire data. The Chi square and t tests were used as needed and p-value of ≤ 0.05 were considered statistically significant. Means were presented ± standard deviation.

## Results

### Characteristics of the study population

The characteristics of patients participating of the study are listed in Table 1. The study population consisted of 101 patients from the five Governorates of Gaza strip (42.6% males and 57.4% females). More than two thirds of patients were from Gaza Governorate, 16.2% from Deir al-Balah (mid-zone) governorate, 8.1% from north Gaza governorate, and about 9.1% from south the governorates (Khan Yunis & Rafah). Their ages ranged from 16 to 76 years, with mean age of 48.8 ± 14.4 years (50.0 ± 13.8 years for males and 48.0 ± 15.0 years for females). The average of body mass index of patients was 27.8 ± 5.2 kg/m^2^ where that of males was 27.2 ± 5.0 kg/m^2^ and that of females was 28.2 ± 5.3 kg/m^2^.

**Table 1.** **Characteristics of the study population.**

Gender (%) – Males – Females	42.4 57.6

Age (years) – Males – Females	48.8 ± 14.4 50.0 ± 13.8 48.0 ± 15.0

Governorate of residence (%) – North Gaza – Gaza City – Mid-zone – Khan Younis – Rafah	8.1 66.7 16.2 5.1 4.0

Body mass index (kg/m^2^) – Males (kg/m^2^) – Females (kg/m^2^) – Obese (%) – Overweight (%) – Normal weight (%) – Underweight (%)	27.8 ± 5.2 27.2 ± 5.0 28.2 ± 5.3 33.7 29.7 32.7 4.0

Type of disease (%) – Thromboembolic events **–** Cardiovascular events **–** Both	50.5 41.4 8.1

Average of INR	2.4 ± 1.5

Bleeding (%) – Major event – Minor event – None	7 38.5 54.5

The patients were taking warfarin treatment because of thromboembolic events (50.5%), cardiovascular disease (41.4%) or both (8.1%). It is worthy mentioning that 51.5% of patients were complaining from other chronic diseases as well. The average of INR of patients was 2.4 ± 1.5 (Table 4.1). There were 45.5% of patients suffering from warfarin-associated adverse effect and 54.5% were not.

### *CYP2C9* & *VKORC1* genotype & allelic frequency

Only one patient (1%) was homozygote for the CYP2C9*2 allele and 25 patients (24.8%) were heterozygotes. The CYP2C9*3 allele was not detected and thus the remaining 74.2% of the study population were homozygotes for the wild-type allele (CYP2C9*1) at the two polymorphic SNPs (Table 2). The calculated frequency of CYP2C9*1 allele is 86.4% and of CYP2C9*2 allele is 13.6%, and both alleles are in Hardy–Weinberg equilibrium (p-value = 0.473). The genotypes for *VKORC1–*1639G > A were 48.5% (GA), 21.8% (AA) and 29.7% (GG). Frequency of the susceptibility allele (A) was calculated to be 46.5% and that of the wild-type allele (G) was 53.5%, and both alleles are in Hardy–Weinberg equilibrium (p-value = 0.8). Different allelic combinations of both genes were obtained (Table 2).

**Table 2.** **Distribution of *CYP2C9* and *VKORC1* genotypes.**

	**Genotype**	**Frequency**	**Percent**	**Allelic frequency**
*CYP2C9*	1*/1*	75	74.3	Allele (*1) = 86.4% Allele (*2) = 13.6%

	1*/2*	25	24.8	

	2*/2*	1	1.0	

	Total	101	100.0	

*VKORC1*	GG	30	29.7	Allele (G) = 53.5% Allele (A) = 46.5%

	GA	49	48.5	

	AA	22	21.8	

	Total	101	100.0	

*CYP2C9/VKORC1*	*1*1/GG	22	21.8	

	*1*1/GA	35	34.7	

	*1*1/AA	18	17.8	

	*1*2/GG	7	6.9	

	*1*2/GA	14	13.9	

	*1*2/AA	4	4.0	

	*2*2/GG	1	1.0	

	Total	101	100.0	

### Warfarin therapy

By comparing the empirically adjusted warfarin dose for all participants (mean = 42.8 ± 15.0 mg/week) to that predicted by the IWPC PGx algorithm (mean = 35.9 ± 69.6 mg/week), 41.6% of the patients were found to receive empirically adjusted doses that are higher than predicted. Among those, 54.8% suffered at least one bleeding event compared with only 35.7% of those having equal or lower than predicted doses (Table 3). The relationship between bleeding events and warfarin dose is statistically significant as represented in Table 3 (p-value = 0.043; Chi-Square = 6.285).

**Table 3.** **Bleeding complications.**

**Bleeding event**	**Given therapeutic dose (mg/week)^†^**			**p-value**

	***Higher than calculated***	***Same as calculated***	***Lower than calculated***	
None	19 (34.5%)	25 (45.5%)	11 (20.0%)	0.043

Yes	23 (52.3%)	19 (43.2%)	2 (4.5%)	

Total	42 (42.4%)	44 (44.4%)	13 (13.1%)	

^†^The values are represented as frequency (valid percent). Data for two genotyped patients were missing. The three values were calculated using the IWPC algorithm.

### Relationship between genotype & WSD

Only 47 patients were found to take WSD and therefore, considered for further analysis of performance of the prediction models. Table 4 compares the mean empirically adjusted WSD according to *CYP2C9* and *VKORC1* genotypes. WSD was lower in case of homo- or heterozygosity for either or both genes. The difference was not statistically significant between CYP2C9*2 allele heterozygotes and the wild-type allele homozygotes (p-value = 0.520). On the other hand, the WSDs were significantly lower for patients carrying one or two copies of the *VKORC1-A* allele compared with homozygotes for the wild-type -*G* allele (p-value = 0.004). The difference was also statistically significant between poor responders (*1*1/GG, *1*2/GA, and *1*1/GA) and good responders (*1*2/GG, *1*1/AA and *1*2/AA) to warfarin (p-value = 0.010).

**Table 4.** **Relation between genotype and warfarin stable dose.**

**Gene(s)**	**Alleles**	**N**	**Given warfarin WSD (mg/week)**		**p-value^†^**

			***Mean***	***Std. Dev.***	
*CYP2C9*	1*/1*	34	42.6	13.8	0.520

	1*/2*	13	39.4	17.5	

	Total	47	41.7	14.8	

*VKORC1*	GG	15	51.2	15.9	0.004

	GA	23	39.1	13.0	

	AA	9	32.5	8.8	

	Total	47	41.7	14.8	

*CYP2C9/VKORC*	*1*1/GG *1*2/GA *1*1/GA	31	45.6	14.6	0.010

	*1*2/GG *1*1/AA *1*2/AA	16	34.1	12.4	

	Total	47	41.7	14.8	

^†^The independent samples t-test was applied for comparing CYP2C9 and CYP2C9/VKORC groups; and the one-way ANOVA was applied for VKORC1 gene. The statistical power, computed using alpha = 0.05, is 0.796.

ANOVA: Analysis of variance; WSD: Warfarin stable dose.

### Performance of three dose-prediction models

**Figure 1.**
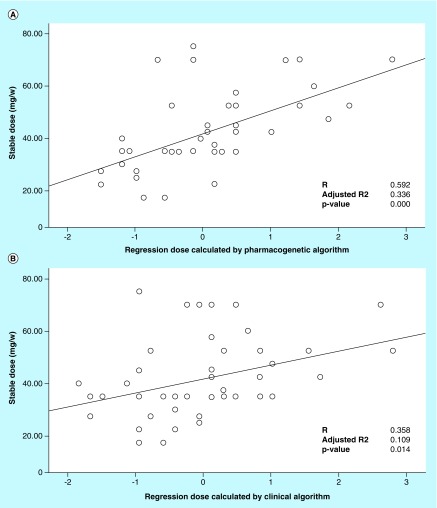
**Linear regression analysis of warfarin stable dose versus predicted dose.** A scatter-plot of the correlation and regression between the empirically adjusted WSD and that predicted using **(A)** the PGx algorithm and **(B)** the clinical algorithm. Please note that some points have identical values and thus are superimposed in the graph, and therefore are darker. PGx: Pharmacogenetic algorithm; WSD: Warfarin stable dose.

Comparisons of three models for dose prediction in the study cohort are summarized in Table 5. The PGx algorithm (MAE = 8.9) provided dose estimates that were closer to empirically reached WSD than estimates derived by the clinical algorithm (MAE = 10.4) and a fixed-dose approach (MAE = 11.1). In general, there was a statistically significant correlation between the empirically reached WSD, and the pharmacogenetically predicted dose (R^2^ = 0.350; p-value = 0.00), and to a lesser extent the clinically calculated (R^2^ = 0.128; p-value = 0.014). The correlation was better when the *CYP2C9* and *VKORC1* genotype was included (Table 5 & Figure 1).

**Table 5.** **Comparison of three dose-prediction models.**

**Model**		**PGx**	**Clinical**	**Fixed-dose**
MAE ± SE (mg/week)		8.9 ± 1.4	10.4 ± 1.4	11.1 ± 1.7

Regression	*R*	0.592	0.358	Not applicable

	R^2^	0.350	0.128	

	*F*	24.233	6.611	

	p-value	0.000	0.014	

MAE: Mean absolute error; PGx: Pharmacogenetic algorithm; SE: Standard error.

The predicted warfarin doses were classified into three groups based on their deviation from the empirically reached WSD. As represented in Table 6, 68.1% of the values calculated with the PGx algorithm were ideal, compared with 53.2% for the clinical algorithm and 44.7% in case of fixed calculation. The NNG for PGx algorithm versus the clinical or fixed-dose algorithms was low (6.7 and 4.3 respectively)

**Table 6.** **Number needed to genotype analysis for pharmacogenetic algorithm versus clinical and fixed-dose algorithms.**

**Model**	**PGx**	**Clinical**	**Fixed-dose**
Within 20% of WSD (ideal)	32 (68.1%)	25 (53.2%)	21 (44.7%)

<20% of WSD (underestimation)	12 (25.5%)	12 (25.5%)	15 (3.9%)

>20% of WSD (overestimation)	3 (6.4%)	10 (21.3%)	11 (23.4%)

Total	47	47	47

ER	0.319	0.468	0.553

ARR (PGx vs clinical)	0.149		

ARR (PGx vs Fixed-dose)	0.234		

NNG (PGx vs clinical)	6.7		

NNG (PGx vs Fixed-dose)	4.3		

ARR: Absolute risk reduction; ER: Event rate; NNG: Number needed to genotype; WSD: Warfarin stable dose.

Event rate (ER): (Underestimation + overestimation)/total number of patients

Absolute risk reduction (ARR) = |*ER_PGx_ - ER_clinical_*|

Number needed to Genotype (NNG): *1/ARR*

## Discussion

Warfarin therapy, the major anticoagulation therapy, is limited by interindividual variation affecting its initiation and maintenance doses [17,24]. As a complex phenotype, both genetic and nongenetic factors are associated with prolonged time to maintenance dose with proper target INR [25–27]. Patients carrying one or two copies of the CYP2C9*2, CYP2C9*3 or *VKORC1* −1639G > A polymorphic alleles are more sensitive to warfarin and require lower doses than those carrying the wildtype alleles [28]. To the best of our knowledge, no study has previously evaluated the Palestinian population for factors affecting warfarin dosing. Therefore, in this study, we determined the frequency of two *CYP2C9* variants (*2 and *3) and one *VKORC1* variant (-1639G > A) in a cohort of Palestinian patients treated with warfarin, and evaluated the use of the IWPC algorithm for estimation of warfarin maintenance dose among the same patients.

The calculated frequency of CYP2C9*2, *3 and *VKORC1–*1639 G > A alleles in our population is 13.6, 0.0 and 46.5% respectively. In comparison, the frequency of the three alleles is respectively 13.3, 2.3 and 42.4% in Saudi Arabia [29] and 15.4, 7.8 and 52.4% in Lebanon [30]. In other native Palestinians, in Israel the frequencies were 21, 7 and 58% in Moslems, and 12, 6 and 53% in Druze [31]. The average worldwide frequency of the CYP2C9*2 allele is 5% ranging from 0% in east Asian to 12% in European populations [32]. Similarly, the average worldwide frequency of the CYP2C9*3 allele is also 5%, ranging from 0% in Africans to 11% in south Asian populations [33]. However, the average worldwide frequency of the *VKORC1–*1639 A-allele is higher (36%), with a minimum of 5% in African and a maximum of 88% in east Asian populations [34].

Presence of the *VKORC1–*1639 G > A allele was found to be significantly associated with higher sensitivity to warfarin therapy in our population. This is evident from requirement of a lower WSD in case of homo- or heterozygous genotypes (Table 3). The same polymorphism was previously shown to be associated with decreased warfarin dosing in white people, African Americans and people of Asian descent [35–38]. On the other hand, the difference in maintenance warfarin dose was not statistically significant between *CYP2C9* *1 and *2 alleles. This is probably due to the low number of *2 allele homozygotes and absence of *3 allele carriers in our population. As we mentioned earlier, the *3 allele is believed to be twice more influential in impairing S-warfarin metabolism than *2 allele (80–90% for *3 compared with 30–40% for *2) [10]. The *VKORC1* genotype alone was proposed to be sufficient for warfarin dose prediction in a cohort of Egyptian patients [39]. *VKORC1*−1639G > A individually explained the greatest variance in dose in whites than blacks and Asians [40].

Previous studies have shown that genotype-based warfarin dosing significantly improved anticoagulation control in white populations [41]. On the other hand, performance of the genetic algorithm was worse than that of the clinical algorithm in African Americans [42]. Therefore, warfarin dosing algorithms should be evaluated in each respective ethnic population before being recommended [16]. In our study, we compared the PGx based algorithm recommended by the IWPC to the same algorithm but with no genotype data (clinical algorithm) and with a fixed dose of 35 mg/week. The PGx algorithm provided dose estimates that were closer to empirically reached WSDs than estimates derived from the clinical and a fixed-dose algorithm. The PGx algorithm gave the least MAE. The pharmacogenetically estimated doses in most patients (68.1%) had ideal retrospectively pharmacogenetically calculated warfarin doses compared with 53.2% for the clinical algorithm and 44.7% in case of fixed-dose model (Table 6). Furthermore, higher coefficient of determination was obtained in case of the PGx model (R^2^ = 0.350) than in case of the clinical model (R^2^ = 0.128; Table 5 and Figure 1). Like many other populations [23,43–47] our data highlights the ability of IWPC PGx algorithm to predict warfarin initiation and maintenance dose in the Palestinian population. More support can be provided by the fact that in this study, NNG for PGx algorithm versus the clinical algorithm was low (6.7) which is even lower than that in IWPC study (13.2) [23] and other studies as well (e.g., 13.3 in Japanese patients) [48]. The NNG was even lower (4.3) for PGx algorithm versus fixed-dose. As previously established, the lower the NNG, the more effective is the prediction model [49].

Additionally, all patients participating in this study did not reach their target INR before at least 1 month from the start of treatment. Lengthy periods of dose adjustment are frequently experienced with patients starting warfarin therapy. During these periods, they become exposed to a high risk for bleeding and thromboembolism. In our study, the occurrence of at least one bleeding event was significantly higher among patients with higher warfarin dose than predicted by the IWPC algorithm compared with those having equal or lower than predicted doses (p-value = 0.027; Table 3).

The outcomes of the study may be limited by the small number of patients with stable warfarin dose (SWD) at the time of the study. We could not enroll more patients mainly because of the lack of their clinical data.

## Conclusion

The relatively common *VKORC1-A* allele is significantly associated with lower WSD in our population. While CYP2C9*3 allele was not detected, the effect of CYP2C9*2 allele (frequency = 13.6%) alone in lowering the WSD is not statistically significant.

The IWPC PGx algorithm for estimation of initial warfarin dose was found reliable in our population, and thus recommended over the empirical approach for adjustment of warfarin dose in indicated patients.

## Future perspective

Although, the correlation between the IWPC algorithm and the WSD was very good, it was not perfect (R = 0.592). This may indicate the presence of other determinants for the individual WSD. Therefore, more research is recommended to evaluate the role of other alleles of *CYP2C9* and *VKORC1* as possible determinants of WSD. Furthermore, more research is recommended involving a larger cohort of patients to establish a Palestinian population based algorithm for prediction of warfarin dose.

**Summary poimts**

**Background**

- The CYP2C9*2, CYP2C9*3 or *VKORC1*−1639G > A polymorphic alleles increase the individual's sensitivity to warfarin.
- Pharmacogenetic algorithms have been established to predict the proper warfarin stable dose based on these allele genotype.
- The main aim of the study was to determine the frequency of *CYP2C9* variants (*2 and *3) and *VKORC1* variant (-1639G > A), and to evaluate the ability of the international warfarin pharmacogenetics consortium (IWPC) algorithm to predict the proper warfarin stable dose in a group of Palestinian patients of Gaza strip.

**Methods**

- Warfarin dose predictions were retrospectively determined for a cohort of Palestinian patients under warfarin therapy.
- *CYP2C9* and *VKORC1* genotypes were determined and used beside other clinical and anthropometric variables to predict the doses using three models (the IWPC pharmacogenetic algorithm (PGx), a clinical algorithm and a fixed-dose calculation of 35 mg/week).
- The performance of the three models was assessed using different statistical analyses.

**Results**

- CYP2C9*2, *3 and *VKORC1-A* alleles frequency in our population is 13.6, 0.0 and 46.5% respectively.
- Carriage of at least one *VKORC1-A* allele significantly reduced the warfarin stable dose (p-value = 0.004).
- The IWPC PGx is more reliable than both the clinical algorithm and the fixed-dose algorithm in predicting the warfarin stable dose in the Palestinian population.
- Occurrence of at least one bleeding event was significantly higher among patients with higher warfarin dose than predicted by the IWPC algorithm compared with those having equal or lower than predicted doses.

**Conclusion**

- The IWPC PGx algorithm can be reliably applied in identifying Palestinian patients who require a low dosage of warfarin and are at greater risk of bleeding complications.
